# Diagnosis, evaluation, and management of cognitive disorders in Parkinson's disease: Consensus recommendations from a modified Delphi process

**DOI:** 10.1002/dad2.70152

**Published:** 2025-07-24

**Authors:** Dana Pourzinal, Deborah Brooks, Deepa Sriram, Leander K. Mitchell, Nancy A. Pachana, Kirstine Shrubsole, Brian Wood, John D. O'Sullivan, Rodney Marsh, Alexander Lehn, Jacki Liddle, Edwin C. K. Tan, W. Kim Halford, Neil Page, Nadeeka N. Dissanayaka

**Affiliations:** ^1^ UQ Centre for Clinical Research The University of Queensland Herston Queensland Australia; ^2^ School of Psychology The University of Queensland Saint Lucia Queensland Australia; ^3^ Queensland Aphasia Research Centre The University of Queensland Herston Queensland Australia; ^4^ Metro South Movement Disorders Service Redland Hospital Brisbane Queensland Australia; ^5^ Griffith University Brisbane Queensland Australia; ^6^ Department of Neurology Royal Brisbane & Women's Hospital Herston Queensland Australia; ^7^ Department of Neurology Princess Alexandra Hospital Brisbane Queensland Australia; ^8^ School of Biomedical Sciences Queensland University of Technology Brisbane Queensland Australia; ^9^ School of Health and Rehabilitation Sciences The University of Queensland Saint Lucia Queensland Australia; ^10^ Department of Occupational Therapy Princess Alexandra Hospital South Brisbane Queensland Australia; ^11^ School of Pharmacy Faculty of Medicine and Health The University of Sydney Sydney New South Wales Australia; ^12^ Kolling Institute Faculty of Medicine and Health The University of Sydney and the Northern Local Health District Sydney New South Wales Australia

**Keywords:** cognitive impairment, dementia, guidelines, Parkinson's disease

## Abstract

**INTRODUCTION:**

Variations in clinical management of cognitive disorders in Parkinson's disease (PD) can delay diagnosis, treatment, and care. To harmonize clinical practice, we aimed to gain consensus on best practice recommendations for the diagnosis, evaluation, and management of cognitive disorders in PD.

**METHOD:**

Fifty‐eight evidence‐based recommendations were presented to an expert panel (*N* = 29) of Australian PD clinicians and researchers using a modified Delphi approach to gauge agreement. A 5‐point Likert scale was used, with a median score > 4 and inter‐quartile range < 1, indicating satisfactory agreement. Optional written feedback was also collected. A steering committee of clinicians, researchers, and lived experience experts (*N* = 13) revised recommendations based on panel feedback.

**RESULTS:**

Fifty‐one evidence‐based and expert‐endorsed recommendations for the diagnosis, evaluation, and management of cognitive disorders in PD were produced.

**DISCUSSION:**

The recommendations serve as a foundational framework to guide clinical practice for cognitive disorders in PD and improve the provision of care.

**Highlights:**

- Recommendations for cognitive disorders in Parkinson's disease were developed.
- Diagnosis, evaluation, and management of cognitive disorders were explored.
- A modified Delphi approach was used.
- A panel of 29 Australian clinician and/or research experts provided input.
- Fifty‐one evidence‐based and expert‐backed recommendations were developed.

## 1 BACKGROUND

The diagnosis, evaluation, and management of cognitive disorders in Parkinson's disease (PD) vary significantly. In part, this variation is due to inherent heterogeneity in the presentation and progression of cognitive deficits in PD,^1^, ^2^ which necessitates a large degree of patient‐centered care tailored to the individual. However, systematic differences in clinical practice above and beyond patient‐centered care can impact the quality of care. For example, variability in the methodology used to identify cognitive disorders, such as choice of neuropsychological measures and diagnostic criteria, impacts who receives a diagnosis.^3^, ^4^ Furthermore, cognition is not routinely evaluated in all health services.^5^ While some clinics may be able to facilitate in‐house neuropsychological assessments, others fail to even broach the topic of cognitive impairment with patients due to time constraints, limited resources, and/or inadequate staff training.^6^

Consequently, the management of cognitive disorders in people with PD lacks cohesion across health services. Streamlined diagnostic care pathways and increased education of health‐care staff have been suggested by people living with cognitive disorders in PD and their care partners to improve the continuity of their care.^7^ However, current practice does not meet the needs of people with cognitive disorders in PD, who report the feeling of “falling through the gaps” in the health‐care system.^7^ Given that cognitive function is a priority and a determinant of quality of life for people with PD and their care partners,^5^, ^8^ greater efforts must be made to address the health, social, and economic implications of cognitive impairment in clinical settings. PDCogniCare is an Australian project aiming to bridge this gap by developing best practice guidelines for cognitive disorders in PD. The present study reports results from a modified Delphi process to develop evidence‐based and expert‐endorsed recommendations for the diagnosis, evaluation, and management of cognitive disorders in PD for use in clinical settings.

## 2 METHOD

### 2.1 Study design

**FIGURE 1:**
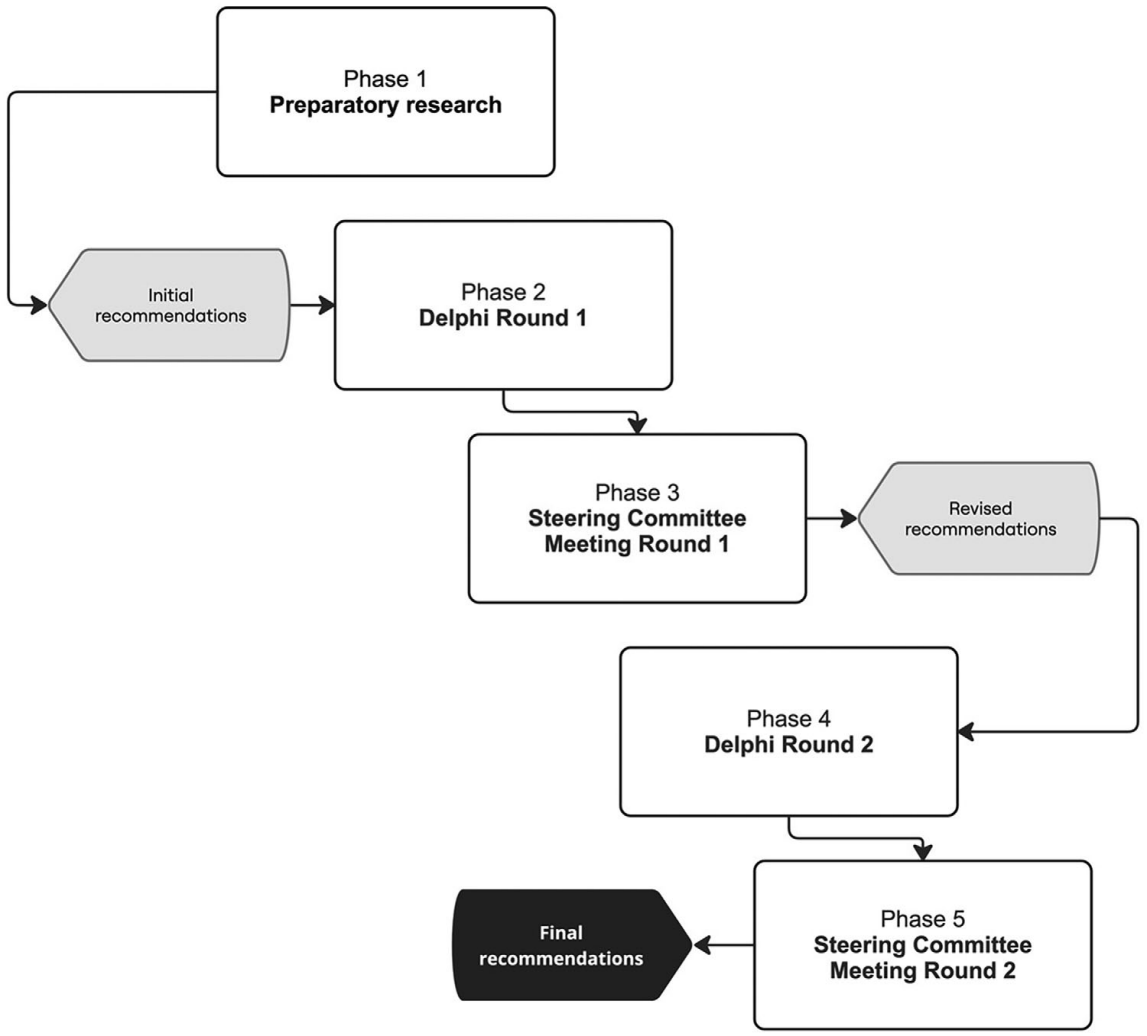
Modified Delphi methodology.

Following Accurate Consensus Reporting Document (ACCORD) guidelines,^9^ a modified Delphi approach was used, in which recommendations were iteratively revised based on two rounds of online survey feedback from a national panel of PD experts to achieve consensus. This methodology was adapted from the national memory clinic guideline development procedure conducted by the Australian Dementia Network (ADNeT),^10^ a PDCogniCare partner. The modified Delphi was used here to identify points of contention and consensus in clinical care for cognitive disorders in PD and to ensure that the final recommendations were endorsed by national experts in the field, to facilitate uptake of the subsequent best practice guidelines in clinics. The Delphi study consisted of five phases (Figure 1). Phase 1 (2023) consisted of preparatory research to produce initial recommendations based on available evidence. In Phase 2 (June 2024), the first round of the Delphi was conducted by presenting the initial recommendations to the expert panel to determine agreement and gather feedback. The Round 1 data were then analyzed and discussed among a steering committee in Phase 3 (August 2024), and revisions were made to the initial recommendations. Round 2 of the Delphi was conducted in Phase 4 (September 2024), during which the revised recommendations were presented back to the expert panel to gauge agreement and gather final feedback. Round 2 data were analyzed and discussed among the steering committee in Phase 5 (October 2024), when the final revisions to the recommendations were made. The PDCogniCare project was approved by the Metro North Health Human Research Ethic Committee (HREC/2023/MNHA/100098). Informed consent was collected from all Delphi panel members.

### 2.2 Selection of Delphi panel and the steering committee

#### 2.2.1 Delphi panel

A sample of at least 20 panel members was sought to achieve adequate representation from the various disciplines involved in PD care.^11^ To achieve this, 133 Australian clinicians and researchers with recognized expertise in the cognitive features of PD were invited to the Delphi panel via e‐mail. Panel invitees were identified through purposive and snowball sampling using the PDCogniCare network, seminal papers in the field, professional societies, special interest groups, and suggestions by other panel members. People with published research and/or extensive clinical experience in the field were considered “experts.” Experts included movement disorders neurologists and nurses, general practitioners, neuropsychologists, psychologists, geriatricians, psychiatrists, neuropathologists, occupational therapists, speech therapists, physiotherapists, and researchers.

#### 2.2.2 Steering committee

A steering committee comprised of 13 multi‐disciplinary experts in PD research and clinical care from the PDCogniCare investigator team was responsible for revisions to the recommendations in line with feedback received from the Delphi panel. This committee included a person living with PD (W.K.H.), care partner (N.P.), geriatrician (B.W.), movement disorders neurologist (J.O.S.), psychiatrist specialized in older persons (R.M.), pharmacist (E.T.), occupational therapist (J.L.), speech pathologist (K.S.), clinical psychologist and clinical neuropsychologist (L.K.M.), clinical psychologist and geropsychologist (N.A.P.), project coordinator and postdoctoral research fellow (D.P.), and chief investigator and professorial research fellow (N.N.D.). Steering committee members were excluded from participation in the Delphi panel.

### 2.3 Preparatory research

Initial recommendations for the diagnosis, evaluation, and management of cognitive disorders in PD were derived from two systematic reviews of literature and a survey of current neuropsychological practices. The first systematic review synthesized all relevant recommendations for cognitive disorders in PD from articles (i.e., guidelines and systematic reviews) published over the past two decades.^12^ The second review evaluated the utility of neuropsychological measures to predict cognitive decline in PD from available longitudinal studies.^13^ A national survey was also distributed to collect information from Australian neuropsychologists regarding current cognitive evaluation methods for people with PD. Findings from the preparatory research were not shared with the Delphi panel to minimize the survey length; however, relevant findings were shared with the steering committee to inform the revision process.

**RESEARCH IN CONTEXT**

- **Systematic Review**: Diagnosis, evaluation, and management of cognitive disorders in Parkinson's disease (PD) varies significantly from clinic to clinic. Disturbances to continuity and quality of care lead to people with PD who experience cognitive symptoms to feel as though they are “falling through the gaps” of health‐care systems.
- **Interpretation**: Our modified Delphi methodology produced 51 recommendations for the diagnosis, evaluation, and management of cognitive disorders in PD. These recommendations were derived from evidence and endorsed by national clinician and/or research experts, with an aim to standardize diagnostic and assessment methodologies, and promote clinical care for cognitive disorders in PD.
- **Future Directions**: The recommendations revealed in the present study are comprehensive, but not exhaustive. Applications of the recommendations should be tailored to the individual, and it is expected that the recommendations will evolve as research in this field progresses. However, the proposed recommendations build a strong foundation for continued work toward harmonizing and optimizing clinical and research practices for cognitive disorders in PD.

### 2.4 Delphi methodology

The Delphi surveys were distributed online via REDCap electronic data capture tools^14^ and included an embedded participant information sheet to obtain electronic informed consent from each respondent. Expert panelists were e‐mailed each round of the Delphi, with a deadline of 1 month for completion and were also offered a $150 gift voucher upon completion of the final round to incentivize participation in all rounds of the Delphi. All panelists completed the surveys independently to prioritize anonymity of the panel. Panel members who participated in Round 1 were invited to complete Round 2, which collected measures of agreement only for recommendations with significant revision. Items that achieved consensus in Round 1 were also included in the Round 2 survey for context and did not require additional ratings of the agreement.

Each recommendation was presented with a 1 to 5 Likert scale (1 = strongly disagree to 5 = strongly agree) to gauge agreement.^11^ An optional text box prompt for written explanations or feedback was provided at the end of each section and the end of the survey. The median and interquartile range (IQR) were calculated to determine which recommendations were contentious.^11^ A median score of > 4 conferred agreement and an IQR of < 1 indicated consensus. Items with low agreement and/or consensus were considered contentious. The item content validity index (I‐CVI) was also calculated, with an I‐CVI > 0.78 indicating high content validity for a given recommendation.^15^ Results from each round were discussed among the steering committee with the objective to remove or revise contentious recommendations (median < 4 or IQR > 1) and consider revising any other recommendations based on the written feedback. The steering committee met exclusively via videoconferencing, and absentees were permitted to provide feedback via e‐mail to be discussed during the meetings.

### 2.5 Key definitions

Key terms were operationalized to standardize terminology used throughout the Delphi process. Brief measures of global cognition, such as the Montreal Cognitive Assessment or Mini‐Mental State Examination, were labelled “brief cognitive screens.” Comprehensive cognitive testing performed by a registered neuropsychologist was referred to as a “comprehensive neuropsychological assessment.”

## 3 RESULTS

### 3.1 Delphi panelists

In total, *N *= 29 clinicians and research experts from Queensland (47%), New South Wales (20%), Western Australia (13%), Victoria (7%), and South Australia (13%) participated in the panel of both rounds of the PDCogniCare Delphi. An additional *N *= 3 panel members only completed Round 1. Table 1 conveys sample demographics of the Delphi panel. Panel members were from multi‐disciplinary backgrounds, although the highest participation rates were from researchers, neurologists, neuropsychologists, and registered nurses. Most respondents were primarily seeing PD patients in the context of either a public hospital or a research clinic.

**TABLE 1:** Sample demographics of Delphi panel.

Variable	Number (*N*)
**Profession**	
Neuropsychologist	6
Clinical psychologist	1
Geriatrician	3
Neurologist	7
Occupational therapist	1
Psychiatrist	1
Registered nurse	6
Researcher	4
**Clinical setting**	
Public hospital	15
Private hospital	1
Private clinic	3
Research	8
Other	2

### 3.2 Delphi process

Preparatory research informed 57 recommendations across the following domains: (1) who should receive a neuropsychological assessment, (2) diagnosis, (3) evaluation, and (4) post‐diagnostic care. These recommendations were circulated to the expert panel for Round 1 of the Delphi, which was open for 4 weeks in June 2024. Round 1 identified seven recommendations with low agreement (median < 4) and/or low consensus (IQR > 1) that were required to be revised or removed for Round 2, and seven other recommendations with acceptable agreement but low consensus (i.e., a potential point of contention) that were considered for revision. Of these, 6 recommendations were removed and 11 were revised during the steering group meeting in August 2024. The 11 revised recommendations were circulated in Round 2, which was open for 4 weeks in September 2024. Round 2 identified 1 recommendation with low agreement that was required to be revised or removed and 10 recommendations with acceptable agreement and consensus; 7 of which were considered for revision due to suggestions proposed within the written feedback. In the second steering group meeting in October 2024, 1 recommendation was revised significantly, and minor word changes were made to 7 recommendations.

### 3.3 Summary of recommendations

In total, 51 recommendations for the diagnosis, evaluation, and management of cognitive disorders in PD reached consensus using the modified Delphi approach. The median, IQR, and ICV‐I for each of the final recommendations are provided in Table 2, as well as the original version of any recommendations that were modified through the Delphi process. Table S1 in supporting information details each of the recommendations that were discarded, the reason for their removal, and their median, IQR, and ICV‐I. Qualitative feedback from Rounds 1 and 2 is also provided in Table S2 and Table S3 in supporting information, respectively.

**TABLE 2:** Final recommendations derived from the modified Delphi process.

Domain	Theme	Final recommendation	Decision	Original version
**Who should receive a cognitive evaluation**	Initial screen	People with PD and their care partners should be asked to report subjective cognitive decline (i.e., perceived problems with memory or thinking) at the time of PD diagnosis.	Accepted after round 1: median = 5; IQR = 1; I‐CVI = 0.89	
		Consider a brief global cognitive screen (e.g., Montreal Cognitive Assessment) for all people with PD at the time of diagnosis as a baseline measure of cognition, regardless of subjective cognitive decline.	Accepted after round 1: median = 5; IQR = 1; I‐CVI = 0.89	
	Follow‐up screen	All people with PD and their care partners should be asked to report subjective cognitive decline (i.e., perceived problems with memory or thinking) at clinical review at least every 12 months.	Accepted after round 1: median = 4; IQR = 2; I‐CVI = 0.86	
		If subjective cognitive decline is reported at a follow‐up appointment, administer a brief global cognitive screen (i.e., Montreal Cognitive Assessment).	Accepted after round 1: median = 5; IQR = 1; I‐CVI = 0.93	
		People with PD who show normal global cognitive function on a brief screen (e.g., Montreal Cognitive Assessment ≥ 27) and report subjective cognitive decline should be considered for a repeat brief cognitive screen within 12 months.	Accepted after round 1: median = 4; IQR = 1.5; I‐CVI = 0.86	
	Initial comprehensive assessment	Comprehensive neuropsychological assessment should be considered for people with PD who show reduced global cognitive function on a brief screen (e.g., individuals with a Montreal Cognitive Assessment score between 20 and 26, inclusive).	Accepted after round 2: median = 4; IQR = 0; I‐CVI = 0.90	People with PD who show reduced global cognitive function on a brief test (e.g., Montreal Cognitive Assessment 20 ≤ 26) should be referred for comprehensive cognitive testing.
		People with PD who show significant impairment in global cognitive function on a brief screen (e.g., Montreal Cognitive Assessment ≤ 19) may be less likely to tolerate a comprehensive neuropsychological assessment. Carefully consider the purpose and overall benefit of assessment in these cases.	Revised after round 2: median = 3; IQR = 2; I‐CVI = 0.62	Any person with PD who shows significant impairment in global cognitive function on a brief test (e.g., Montreal Cognitive Assessment ≤ 19) does not require comprehensive cognitive testing.
		If significant impairment in global cognitive function on a brief test (e.g., Montreal Cognitive Assessment ≤ 19) and functional impairment is present, clinically assess for a diagnosis of dementia.	Accepted after round 1: median = 4; IQR = 1; I‐CVI = 0.86	
	Follow‐up comprehensive assessment	People with PD who are diagnosed with mild cognitive impairment after comprehensive neuropsychological assessment should be considered for a repeat comprehensive assessment within 12 months.	Accepted after round 1: median = 4; IQR = 2; I‐CVI = 0.82	
		People with PD who are diagnosed with mild dementia after a comprehensive neuropsychological assessment should be scheduled for a repeat comprehensive neuropsychological assessment at the discretion of the clinician, the person with PD, and their care partners.	Accepted after round 1: median = 4; IQR = 1; I‐CVI = 0.89	
		People with PD who are diagnosed with moderate to severe dementia after a comprehensive neuropsychological assessment are less likely to tolerate or benefit from a repeat comprehensive neuropsychological assessment. Consider a needs assessment to guide care.	Accepted after round 2: median = 4; IQR = 1; I‐CVI = 0.90	People with PD who are diagnosed with moderate to severe dementia after comprehensive cognitive testing should not receive a repeat comprehensive cognitive assessment.
	Indications for cognitive evaluation	People with PD should be considered for a comprehensive neuropsychological assessment: If subjective cognitive decline or decline in functional ability is perceived by the person with PD or the informant If subjective cognitive decline or decline in functional ability is perceived by the clinician If reduced cognition is demonstrated on a brief global cognitive assessment If behavioral and/or psychological symptoms are present When functional neurosurgery (e.g., DBS, Gamma knife) is being considered When there is diagnostic uncertainty or for the purpose of differential diagnosis Capacity assessment (e.g., for guardianship) is required if the person with PD requests a cognitive evaluation	All accepted after round 1: median = 4–5; IQR = 0–1.5; I‐CVI = 0.86–0.93	
**Diagnosis**	Diagnostic criteria	DSM‐5‐TR criteria for major neurocognitive disorder (dementia) due to PD and mild neurocognitive disorder (mild cognitive impairment) due to PD should be used to diagnose cognitive disorders.	Accepted after round 1: median = 4; IQR = 0; I‐CVI = 0.82	
		Objective decline should be defined as > 1 SD decrease (based on relevant norms) in performance from previous testing, or > 1 SD decrease from estimated premorbid levels where previous testing is unavailable.	Accepted after round 1: median = 4; IQR = 1; I‐CVI = 0.82	
		The following tests may be used to explore alternative causes of cognitive impairment before a diagnosis of dementia in PD: Psychiatric evaluation (depression, anxiety, hallucinations) Blood tests (vitamin B12, folate, TSH, liver function, anti‐neuronal antibody panel, syphilis, zoonoses, and HIV) MRI or CT (vascular dementia, stroke, and brain tumors) Fluorodeoxyglucose positron emission tomography (cortical hypometabolism)	All accepted after round 1: median = 4; IQR = 1; I‐CVI = 0.75–0.86	
		The following tests may be used to rule out alternative causes of cognitive impairment prior to a diagnosis of mild cognitive impairment in PD: Psychiatric evaluation (depression, anxiety, hallucinations) Blood tests (vitamin B12, folate, TSH, liver function, anti‐neuronal antibody panel, syphilis, zoonoses, and HIV) MRI or CT (vascular dementia, stroke and brain tumors)	All accepted after round 1: median = 4; IQR = 1–1.25; I‐CVI = 0.75–0.86	
		Where applicable, cognitive subtypes (e.g., amnestic, frontal dysfunction) should be identified to inform care procedures (e.g., tailored cognitive rehabilitation), psychoeducation, and entry into clinical trials.	Accepted after round 1: median = 4; IQR = 0.75; I‐CVI = 0.93	
	Delivery of diagnosis	Diagnosis of cognitive disorders should be clearly communicated verbally to people with PD and their care partners, with written information provided for personal review.	Accepted after round 1: median = 5; IQR = 0; I‐CVI = 1.00	
		Diagnosis of cognitive disorders should be delivered to people with PD in a sensitive and empathetic manner.	Accepted after round 1: median = 5; IQR = 0; I‐CVI = 1.00	
	Consent and confidentiality	People with PD who receive a cognitive diagnosis should be asked if and with whom the outcome of their assessment may be shared.	Accepted after round 1: median = 5; IQR = 0; I‐CVI = 1.00	
		Neuropsychological test results should be shared with the primary care physician (e.g., GP) and any other allied health service that may benefit from this information (e.g., speech pathology, physio).	Accepted after round 1: median = 5; IQR = 1; I‐CVI = 0.96	
**Evaluation**	Neuropsychological assessment toolkit	Due to its greater sensitivity to mild cognitive impairments in PD, the Montreal Cognitive Assessment is the preferred brief test of global cognition in PD.	Accepted after round 1: median = 4; IQR = 1; I‐CVI = 0.86	
		Consider self‐report tools, informant interview, observational evaluation and formal testing for assessment of activities of daily living to determine functional impairment due to cognitive impairment rather than motor impairment.	Accepted after round 2: median = 4; IQR = 1; I‐CVI = 0.93	An assessment of instrumental activities of daily living should be administered to determine functional impairment due to cognitive impairment, with care taken to parse out impairment due to motor impairment (e.g., using cognitive sub‐score of the Pfeiffer Functional Activities Questionnaire).
		Neuropsychological test batteries to identify cognitive disorders in PD should be designed to minimize duration of the test battery to prevent fatigue and to mitigate influence of motor symptomatology on the test results.	Accepted after round 1: median = 4.5; IQR = 1; I‐CVI = 0.93	
		The following tests are recommended for cognitive evaluations in PD due to their sensitivity to cognitive decline in PD, reasonable duration, availability of alternative forms, and limited interference of motor symptoms: **Verbal Memory**: Rey Auditory Verbal Learning Test, California Verbal Learning Test, Hopkins Verbal Learning Test **Visual Memory**: Rey–Osterrieth Complex Figure Test, Brief Visuospatial Memory Test **Executive Function**: Trail‐Making Test Part B, STROOP Word–Color scale **Attention/processing speed**: Symbol Digit Modalities Test, Trail‐Making Test Part A, STROOP Word or Color scales **Visuospatial function**: Visual memory “copy” trial, Pentagon copying **Fluency**: Category fluency	Accepted after round 1: median = 4; IQR = 0–1; I‐CVI = 0.46–0.68	
	Preparing for the assessment	Prior to the neuropsychological assessment, people with PD and their care partners should receive verbal and written information regarding the context and purpose of their assessment to prepare for the appointment.	Accepted after round 2: median = 4; IQR = 1; I‐CVI = 0.93	Added after round 1.
		Referral to an experienced psychologist should be considered if people with PD or their care partner express emotional distress during the waiting time for the neuropsychological assessment and require support	Accepted after round 2: median = 4; IQR = 1; I‐CVI = 1.00	Added after round 1.
	Assessment procedures	Efforts to stabilize affective, mood, or psychiatric conditions should be made prior to cognitive evaluation, as this may influence test administration and results.	Accepted after round 1: median = 4; IQR = 1; I‐CVI = 0.93	
		People with PD should be tested in the ON state (i.e., when Parkinsonian symptoms are controlled), unless specific OFF state testing is required.	Accepted after round 1: median = 4; IQR = 1; I‐CVI = 0.89	
		Where possible, consider aligning comprehensive neuropsychological assessment with antiparkinsonian medication regimes to ensure that evaluations are conducted during the ON state.	Accepted after round 2: median = 4; IQR = 1; I‐CVI = 0.97	People with PD should be instructed to take their dopaminergic medication 30 minutes to 1 hour prior to neuropsychological assessments to induce the ON state.
		Telehealth neuropsychological assessments should be made available to people with PD who cannot access neuropsychology services in person.	Accepted after round 1: median = 4.5; IQR = 1; I‐CVI = 0.96	
		People with PD of culturally and linguistically diverse backgrounds should have access to interpreters and/or translated versions of neuropsychological assessments when possible to facilitate assessment and minimize cultural bias.	Accepted after round 1: median = 5; IQR = 0; I‐CVI = 0.96	
	Feedback sessions	A feedback session should be offered to people with PD and their care partners within 1 month of their neuropsychological assessment to receive any formal diagnoses and discuss a care plan.	Accepted after round 1: median = 5; IQR = 1; I‐CVI = 1.00	
		Feedback of neuropsychological assessment results should be delivered with sensitivity and appropriate consent.	Accepted after round 2: median = 5; IQR = 0; I‐CVI = 0.97	People with PD should receive high‐level feedback on their cognitive performance (e.g., “above average,” “average,” “below average” performance within cognitive domains).
**Post‐diagnostic care**	Pharmacological treatments	The following drugs could be considered in the treatment of dementia in PD: First line: rivastigmine (transdermal in preference to oral), donepezil Second line: galantamine, NMDA antagonists (memantine)	First line accepted after round 1: Median = 4–5; IQR = 1; I‐CVI = 0.36 Second line revised after round 1: median = 3; IQR = 1–1.5; I‐CVI = 0.32–0.29	
		Cautious deprescribing of medications should be considered after diagnosis of any cognitive disorder in PD, including: Anticholinergics (e.g., tolterodine, oxybutynin, tricyclic antidepressants) Benzodiazepines (e.g., alprazolam, diazepam) Antipsychotics with high affinity for D2 receptors (e.g., haloperidol, risperidone)	Accepted after round 2: median = 5; IQR = 1; I‐CVI = 0.59	Discontinuation of the following drugs should be considered after diagnosis of any cognitive disorder in PD: Anticholinergics (e.g., tolterodine, oxybutynin) Benzodiazepines (e.g., alprazolam, diazepam) Amantadine Selegiline Antipsychotics with high affinity for D2 receptors (e.g., haloperidol, risperidone) Tricyclic antidepressants
		For people with cognitive disorders in PD, antipsychotics should be prescribed at the lowest dose for the shortest possible time, reassessing their need every 12 weeks.	Accepted after round 1: median = 4; IQR = 1.5; I‐CVI = 0.36	
		Polypharmacy should be minimized where possible for people with cognitive disorders in PD.	Accepted after round 1: median = 5; IQR = 0; I‐CVI = 0.39	
	Non‐pharmacological treatments	The following non‐pharmacological treatments for people with PD who receive any cognitive diagnosis should be considered: Goal‐focused rehabilitation (e.g., assistive technology, home modifications) Cognitive rehabilitation (cognitive exercises) Cognitive rehabilitation (computerized cognitive training) Activities of daily living training/support Memory strategy training Dance exercise Music contingent gait training Tai chi	All accepted after round 1: median = 4; IQR = 1; I‐CVI = 0.75‐0.93	
	Lifestyle modifications	The following lifestyle modifications should be discussed with people with PD who receive any cognitive diagnosis and their care partners: Community mobility and home safety strategies Falls prevention strategies Physical activity Cognitive stimulation (e.g., puzzles, crosswords) Social engagement Mediterranean diet	All accepted after round 1: Median = 4–5; IQR = 1; I‐CVI = 0.89–1.00	
		People with PD with a cognitive diagnosis should be advised to: Take their time while completing tasks Let their support network know if they are having trouble Seek help if depressed or anxious Develop a highly structured daily routine to follow Develop cognitive coping strategies (e.g., for attention, memory) with occupational therapists or psychologists	All accepted after round 1: median = 4–5; IQR = 1; I‐CVI = 1.00	
	Care services	People with PD should receive advice on or referral to the following health‐care services after receiving any cognitive diagnosis: Non‐governmental organizations (e.g., Dementia Australia)DieticianOccupational therapistOld age psychiatristPhysiotherapist PsychoeducationPsychologistSocial workerSpeech therapistExercise physiologist	All accepted after round 1: median = 4; IQR = 1–2; I‐CVI = 0.93–1.00	
		Health‐care services for cognitive impairment (e.g., psychoeducation, allied health) should also be offered to people with PD with only subjective cognitive decline.	Accepted after round 1: median = 4; IQR = 1; I‐CVI = 0.86	
	Post‐diagnostic care plan	A detailed post‐diagnostic care plan should be developed in partnership with people with PD and their care partners soon after a cognitive diagnosis and monitored during follow‐up appointments.	Accepted after round 1: median = 4; IQR = 1; I‐CVI = 0.96	
		The post‐diagnostic care plan should be shared with people with PD and their care partners and include links to online resources and contact details for relevant support services (e.g., psychoeducation, support groups, counseling, legal aid).	Accepted after round 1: median = 4; IQR = 1; I‐CVI = 1.00	
		The primary care physician (e.g., GP) should be involved in the implementation of the post‐diagnostic care plan where possible.	Accepted after round 1: median = 5; IQR = 1; I‐CVI = 1.00	
	Important discussions	Advance care planning or directives and estate planning should be discussed with people with PD and their care partners soon after diagnosis of PD and reconsidered soon after diagnosis of any cognitive disorder.	Accepted after round 2: median = 5; IQR = 1; I‐CVI = 0.90	Advance care planning or directives should be discussed with people with PD at the time of a dementia diagnosis.
		Sensitive, tailored discussions regarding fitness to drive should be considered for people with PD and their care partners soon after diagnosis of PD and again soon after diagnosis of any cognitive disorder.	Accepted after round 2: median = 5; IQR = 1; I‐CVI = 0.97	Fitness to drive should be assessed for people with PD at the time of a dementia diagnosis.
		Supports and adjustments for people currently working should be discussed with people with PD soon after diagnosis of any cognitive disorder.	Accepted after round 1: median = 4; IQR = 1; I‐CVI = 1.00	
		Community mobility monitoring or planning should be discussed with people with PD and their care partners soon after diagnosis of any cognitive disorder to provide adjustment support for transitions with mobility.	Accepted after round 1: median = 4; IQR = 1; I‐CVI = 0.96	
		People with cognitive disorders in PD and their care partners should be made aware of any local clinical trials and/or relevant local support services (e.g., dementia support groups or support groups for carer partners) of potential relevance.	Accepted after round 1: median = 4.5; IQR = 1; I‐CVI = 1.00	

Abbreviations: DBS, deep brain stimulation; DSM‐V‐TR, Diagnostic and Statistical Manual of Mental Disorders Fifth Edition, Text Revision; GP, general practitioner; HIV, human immunodeficiency virus; I‐CVI, item content validity index; IQR, interquartile range; PD, Parkinson's disease; SD, standard deviation; TSH, thyroid‐stimulating hormone.

## 4 DISCUSSION

The present study revealed 51 evidence‐based and expert‐endorsed recommendations for the diagnosis, evaluation, and management of cognitive disorders in PD. These recommendations integrate more than a decade of novel evidence with clinical expertise to build on previous gold‐standard guidelines for cognitive impairment.^16^, ^17^, ^18^ In particular, the evidence base for non‐pharmacological interventions for cognitive impairment in PD has grown significantly, as well as care considerations for people living with dementia.^12^ The present recommendations account for these advances, providing up‐to‐date advice on post‐diagnostic care for people with cognitive disorders in PD.

The present recommendations also expand on previous guidelines for dementia with Lewy bodies (including PD dementia [PDD]) by providing specific, evidence‐based recommendations for neuropsychological evaluation procedures in PD.^19^ Where previous guidelines did advise on neuropsychological testing in PD,^16^, ^17^ these were based on limited studies.^20^ Since then, ample evidence has been produced exploring the utility and validity of cognitive measures in PD. While many of the recommended measures in the present paper are similar to those endorsed by previous guidelines for PDD and PD‐mild cognitive impairment [MCI],^16^, ^17^ recommendations were based on a thorough systematic review that excluded many previously endorsed measures due to insufficient or weak evidence.^13^ A national survey of neuropsychologists also revealed consistent use of Diagnostic and Statistical Manual of Mental Disorders Fifth Edition, Text Revision (DSM‐5‐TR) criteria for neurocognitive disorder due to PD to make diagnoses, which informed the decision to recommend DSM‐5‐TR criteria over previous gold‐standard criteria for PDD or PD‐MCI.^16^, ^17^

The recommendations are comprehensive but not exhaustive; as emerging areas of research, such as biomarkers and artificial intelligence, continue to grow in the context of PD cognition,^21^, ^22^ so too will the list of recommendations. Moreover, although these recommendations are derived from an Australian expert consensus, they are also readily adaptable to suit international contexts. For example, there are translated versions of many of the recommended neuropsychological measures, and non‐governmental support organizations for PD and dementia exist in many countries, such as Parkinson's UK (UK), Alzheimer's Association (United States), and Associação Brasil Parkinson (Brazil).

There is also much room for adaptability and individualization of the recommendations to meet the unique demands of patient‐centered care. Similarly, implementation of the recommendations will need to be interpreted in the context of each clinic, with adjustments made to suit staffing, funding, and available resources. This is particularly true for certain recommended investigations that may be unavailable outside of metropolitan regions, such as brain imaging, which is increasingly imperative for people with PD who experience cognitive decline. Given the degree of complexity in PD clinical care, this will require robust implementation plans addressing the barriers and facilitators of each clinic (e.g., availability of a movement disorders nurse, on‐site allied health services). Nonetheless, the recommendations provide a strong foundation of knowledge, particularly for clinicians new to the field and from various disciplines.

### 4.1 Clinical implications

People with PD and their care partners have reported that clinical management of cognitive features of PD is “disjointed, non‐specific, and under‐resourced.”^7^ To improve this standard of care and homogenize clinical practices, this study developed a series of evidence‐based and expert‐endorsed recommendations for the diagnosis, evaluation, and management of cognitive disorders in PD. In doing so, we offer guidance to PD clinicians based on current evidence with the aim of streamlining cognitive care to meet patient needs and close the gap between knowledge and practice. The recommendations will inform the development of best practice guidelines with input from lived experience experts to ultimately improve the quality of care and quality of life of people living with PD.

Increased awareness of cognitive impairment in PD will facilitate a proactive rather than reactive approach, allowing care teams to effectively plan for the future. The guidelines will also champion the autonomy of people with cognitive disorders in PD and their care partners, who have reported feeling “left in the dark” due to the complexity of current clinical processes.^7^ This is especially true considering that patients, caregivers, and clinicians alike can struggle to recognize cognitive impairments due to their insidious onset, as functional impairment due to cognitive impairment can be veiled by physical disability, making it difficult to assess.^23^ Discussing dementia can also be challenging for both patients and clinicians due to mutual fears about cognitive decline and the perception that little can be done. However, when cognitive diagnoses are made accurately and sensitively with a clear management plan, it can be a positive force for people with PD and their families, helping them to understand their cognitive and behavioral changes, find support, and plan for the future. However, to ensure that high‐quality care is maintained, future research should aim to empirically evaluate the health and economic impact of these guidelines on an individual and societal level, and provide updates every 5 years based on new literature and feedback from end users.

### 4.2 Strengths and limitations

A considerable strength of the study was the use of a modified Delphi approach. Assembling highly experienced multi‐disciplinary panels produced a broad range of perspectives. Including people with lived experience of PD on the steering committee provided insights from a consumer point of view and allowed for rich discussions about what practices are ideal from clinical, research, and lived experience perspectives. Another benefit of using a Delphi process was that it may serve as a facilitator for the uptake of the final guidelines, as clinicians who contributed to the development of the recommendations may be more likely to accept and use them.

The target sample size for the Delphi panel was achieved, although there was an expected low response rate to invitations due to the heavy workloads of the clinicians and research experts. There were high response rates from the Australian state where the PDCogniCare networks are based (Queensland); however, representation from other Australian states on the Delphi panel helped to account for potential state‐based differences in clinical practice. There was also strong representation from professions primarily involved in the management of the cognitive disorders in PD (i.e., neuropsychologists and neurologists). Furthermore, Delphi panel members were anonymous to one another and thus quantitative and qualitative results of each Delphi round were not susceptible to social biases (e.g., social desirability bias and imbalanced group dynamics). Conversely, the lack of anonymity during steering group meetings may have influenced discussions and decision making through the influence of group dynamics.

The final recommendations are also limited by critical gaps in knowledge, as the current available literature fails to address key issues such as how to measure the unique impact of cognitive symptoms on functional ability, how frequently cognitive screening and follow‐up assessments should occur, and how to manage motor fluctuations for neuropsychological assessments. There was also limited evidence and thus few recommendations for tele‐neuropsychology in PD, which is an emerging field of research catalyzed by the COVID‐19 pandemic that will greatly benefit people with PD in regions with limited access to neuropsychology services. Finally, the present findings did not advise on the diagnosis of dementia with Lewy bodies, particularly in those with an initial diagnosis of PD, although DIAMOND‐LEWY guidelines provide effective guidance on this topic.^19^ As research works toward optimizing neuropsychological practices in PD, updates to the recommendations are advised.

## 5 CONCLUSIONS

This study used a modified Delphi approach to gain consensus on a series of evidence‐based recommendations for the diagnosis, evaluation, and management of cognitive disorders in PD. These recommendations will form the foundations of the PDCogniCare best practice guidelines for cognitive disorders in PD, with the intention of optimizing and harmonizing clinical care and raising awareness among multidisciplinary health‐care workers of the cognitive features of PD.

## CONFLICT OF INTEREST STATEMENT

The authors declare no conflicts of interest. All author disclosures are available in the supporting information.

## CONSENT STATEMENT

All human subjects provided informed consent.

## Supporting information



Supporting Information

Supporting Information

## Data Availability

Data files may be shared, upon request.
