# Supplementary material for: Diagnosis, evaluation, and management of cognitive disorders in Parkinson's disease: Consensus recommendations from a modified Delphi process
Source: Alzheimers Dement (Amst). 2025 Jul 24;17(3):e70152. doi: 10.1002/dad2.70152 (PMC12287750; doi:10.1002/dad2.70152)
Supplement: Supplementary file 1 — Supporting Information [file DAD2-17-e70152-s002.docx]

Supplementary Material

| *eTable 1*. Recommendations removed from the Delphi process | | | | | |
| --- | --- | --- | --- | --- | --- |
| **Whole recommendations removed from Delphi** | | | | | |
| **Recommendation** | | **Reason for removal** | **Median** | **IQR** | **ICV-I** |
| 1 | People with PD should be referred to allied health (e.g., neuropsychology, occupational therapy) for comprehensive neuropsychological evaluation for evidence of cognitive decline. | Removed due to comments that only neuropsychologists should perform comprehensive evaluations | 4 | 2 | 0.96 |
| 2 | People with PD should be considered for comprehensive neuropsychological assessment: When assessment of medical fitness to drive is required | Removed due to OT suggesting medical fitness to drive is separate assessment | 4 | 1.75 | 0.89 |
| 3 | People with speech difficulties (e.g., suspected dysphagia) should receive a diagnostic assessment from a speech pathologist prior to cognitive evaluation. | Removed due to lack of agreement | 3 | 1 | 0.71 |
| 4 | The following drugs should be considered in the treatment of mild cognitive impairment in PD: Rivastigmine | Removed due to lack of evidence for MCI and lack of agreement | 3 | 2 | 0.21 |
| 4 | The following drugs should be considered in the treatment of mild cognitive impairment in PD: Donepezil | Removed due to lack of evidence for MCI and lack of agreement | 3 | 2 | 0.25 |
| 4 | The following drugs should be considered in the treatment of mild cognitive impairment in PD: Galantamine | Removed due to lack of evidence for MCI and lack of agreement | 2 | 2 | 0.18 |
| 4 | The following drugs should be considered in the treatment of mild cognitive impairment in PD: NMDA antagonists (Memantine) | Removed due to lack of evidence for MCI and lack of agreement | 2 | 1.5 | 0.18 |
| 4 | The following drugs should be considered in the treatment of mild cognitive impairment in PD: MAO-B inhibitors (Rasagiline) | Removed due to lack of evidence for MCI and lack of agreement | 3 | 1.5 | 0.21 |
| 4 | The following drugs should be considered in the treatment of mild cognitive impairment in PD: Selective norepinephrine reuptake inhibitors (Atomoxetine) | Removed due to lack of evidence for MCI and lack of agreement | 2 | 1.5 | 0.18 |
| 5 | The following PD treatments should be considered for people who receive a diagnosis of dementia: Intrajejunal levodopa infusion | Removed due to lack of agreement | 3 | 0.5 | 0.29 |
| 5 | The following PD treatments should be considered for people who receive a diagnosis of dementia: Levodopa- carbidopa intestinal gel infusions | Removed due to lack of agreement | 3 | 1 | 0.25 |
| 5 | The following PD treatments should be considered for people who receive a diagnosis of dementia: Subcutaneous apomorphine pump | Removed due to lack of agreement | 2 | 1 | 0.18 |
| 6 | The following PD treatments should be considered for people who receive a diagnosis of MCI: Intrajejunal levodopa infusion | Removed due to lack of agreement | 3 | 1 | 0.32 |
| 6 | The following PD treatments should be considered for people who receive a diagnosis of MCI: Levodopa- carbidopa intestinal gel infusions | Removed due to lack of agreement | 3 | 1 | 0.32 |
| 6 | The following PD treatments should be considered for people who receive a diagnosis of MCI: Subcutaneous apomorphine pump | Removed due to lack of agreement | 3 | 0.5 | 0.32 |
| **Individual items removed from recommendations** | | | | | |
| 14 | The following tests may be used to rule out alternative causes of cognitive impairment prior to a diagnosis of dementia in PD: **Amyloid PET** | Removed amyloid PET due to lack of agreement | 3.5 | 1 | 0.68 |
| 15 | The following tests may be used to rule out alternative causes of cognitive impairment prior to a diagnosis of mild cognitive impairment in PD: **FDG PET** | Removed FDG PET due to lack of evidence for MCI and lack of agreement | 3 | 1 | 0.64 |
| 15 | The following tests may be used to rule out alternative causes of cognitive impairment prior to a diagnosis of mild cognitive impairment in PD: **Amyloid PET** | Removed amyloid PET due to lack of evidence for MCI and lack of agreement | 3 | 1.75 | 0.54 |
| 35 | The following drugs could be considered in the treatment of dementia in PD: **Rasagiline** | Removed due to lack of agreement | 3 | 1.5 | 0.25 |
| 35 | The following drugs could be considered in the treatment of dementia in PD: **Atomoxetine** | Removed due to lack of agreement | 3 | 0.5 | 0.29 |
| 39 | The following non-pharmacological treatments for people with PD who receive any cognitive diagnosis should be considered: **Karate** | Removed due to lack of agreement | 3 | 1 | 0.75 |
| 39 | The following non-pharmacological treatments for people with PD who receive any cognitive diagnosis should be considered: **TCD-S** | Removed due to lack of agreement | 3 | 2 | 0.46 |
| Note. IQR = Inter-Quartile Range, ICV-I = Item Content Validity Index, PD = Parkinson's disease | | | | | |

| *eTable 2*. Written feedback from Round 1 |
| --- |
| **Who should receive a neuropsychological evaluation?** |
| ·       *[Screen at time of diagnosis] - I would think this would be important if pt/family has subjective cognitive concerns. [People who show subjective decline at PD diagnosis] - This would probably warrant consideration of a neuropsychological evaluation, as opposed to a repeat screen by the neurologist in 12 months. [People with moca < 19 do not require comprehensive assessment] - I think this would depend on the referral question. [People with MCI to be retested at 12 months] - I think this should depend on whether there are subjective concerns of worsening.* |
| ·       *I expect a degree of mild cognitive impairment in my patients with PD. If there is more marked impairment early in the condition this makes me suspicious for an atypical parkinsonian disorder. However I rarely pursue comprehensive cognitive assessment as i will use other signs/clues to help with the diagnosis. In more advanced PD if there are concerns regarding cognitive decline i will determine the severity based on history and a MOCA; again rarely proceed to more comprehensive testing* |
| ·       *I do not think it's appropriate to discuss cognitive decline on diagnosis as people may b in shock at receiving a dx of PD, let alone be faced with cognitive impairment or dementia. Some patients may have immediate concerns re dementia or identified cognitive impairment which should be carefully explained. some patients are not ready and do not want to hear about cognitive impairment and this needs to be assessed on a case by case basis. If cognitive impairment is not an identified high priority symptoms at time of consult it can waste resources doing theses tests at each consult and may be unnecesary* |
| ·       *in early parkinsons disease mild cognitive changes often have some impact on daily function but discussion/ education around managing these can be sufficient for some patients, and repeat objective assessment yearly whilst potentialy useful for some patients is not likely to alter management if there is no functional implication for them for some years, and clinic review time may be better spent on managing other symptoms. access to comprehnsive testing and counselling is very important for patients and families noting cognitive changes and functional implications* |
| ·       *I think regardless of severity, cognitive evaluation is valuable (even if briefer rather than comprehensive). Any areas of preservation are useful to highlight in management and for family.* |
| ·       *I have consistently observed clinicians focusing primarily on motor symptoms, with little if any attention given to non-motor symptoms and cognitive functioning. Limited attention to potential cognitive impairment frequently impacts on care with clinicians speaking only with the person with PD and not including carer/family in at least some of the consultations. Also impacts on decision making as to the competence of the person to make fully informed decisions on future treatments.* |
| ·       *Cognitive decline is a critically important symptom of PD and should be appropriately assessed, monitored and treated with similar priority to the motor symptoms of PD.* |
| ·       *If a patient has a history of cognitive symptoms, then when to reasses the patient will be influenced by a number of individual factors e.g., would the patient like to be re-tested. In addition, the other aspects that is important: are the cognitive deficits affecting the pts ability to undertake various ADLs including driving. this affects the clinical decision making process.* |
| ·       *Ideally people with PD should be followed up every 12 months, for physical and cognitive assessments.* |
| ·       *I think formal and regular assessment is important to monitor for decline (there are lots of consequences of deteriorating cognition for the person with PD and their carer/family). This regular assessment can help with care planning (daily as well as financial etc). And potentially reduce carer burden if appropriate interventions are put in place early (before things become overwhelming). If diagnosed with moderate to severe dementia - it may not be that further testing is needed.* |
| ·       *Cognitive symptoms can impact a person's QOL more than the physical symptoms. Many participants and members of our group want to be assessed regularly so that they can psychologically adjust to cognitive decline and put measures in place to support them and their souse/carer.* |
| ·       *No need for repeat testing if dementia; reasons for MCI might be social/psychological stressors so this should be taken into account* |
| ·       *I don't think we need to repeat cognitive testing unless patients or their family expressed concerns or if the treating clinicians have noticed decline during their consultation. If indicated then brief cognitive screen should be repeated. It's also important to be aware of reinforcing beliefs of subjective cognitive impairment for the worried wells and normal age related decline. Ideally treating doctors should be able to interview and assess for factors that may contribute to cognitive complaints before referring patients for repeat comprehensive assessment.* |
| ·       *Cognitive screening should be competed for all PD patients at initial review, with cognitive symptoms. However there is often a lack of resources in general neurology or Mvt clinics to continue to monitor the memory and address these better manage them and hence would suggest geriatrics offer assistances in further assisting with PD cognitive management to improve patient care.* |
| ·       *Whilst cognitive impairment is frequently encountered in PD, the decision to further assess for cognition especially in terms of detailed assessment, will be guided by patient factors as well as the availability of resources. Furthermore, there will be also be a pragmatic notion of 'will the assessment change the management plan?' or is this more 'academic'.* |
| ·       *Questions related to screening instruments- this is a bare minimum that doctors/referrers can do but intact or impaired performance does not necessarily negate a referral for a more comprehensive / targeted neuropsych assessment. They are not sensitive to age and education levels and do not often inform whether there may be an additional pathological process accounting for cognitive changes or the impact of mood on cognition* |
| ·       *Q2:11 and 2:12- repeat assessment is likely needed, particularly if patient or family note change in cognition, behaviour etc.* |
| ·       *I disagree with recommendations 2.11 and 2.12. Even when a dementia diagnosis is present, it is critical to track longitudinal progression of cognitive and functional decline, as this may have important implications for adjusting treatment regimen and improving quality of life.* |
| ·       *It depends how sever "moderate to severe dementia" is in terms of whether there is utility in further serial cognitive testing. Severity may help in prognostication and determining likely need for services.* |
|  |
| **Diagnosis** |
| ·       *Urine test for potential UTRI-related delirium* |
| ·       *For recommendation 3.8, I think that it is critical that this be revised to include "and to a family member, friend or other care provider," in order to ensure that information is appropriately communicated and understood, particularly when cognitive impairment is present for the individual with PD. For recommendation 3.10, I think that there is a burden to share the information with a family member, friend or other care provider, for the reasons communicated above (i.e. making sure that information has been understood and that an appropriate care plan can be formulated and actioned. The individual with PD themselves may not have the capacity to be solely responsible for this* |
| ·       *For 3.3 - I would have thought <1SD from premorbid estimates* |
| ·       *I'm not sure I understand this question - only people with PD who consent should receive feedback on their neuropsychological test performance. Surely they have consented to neuropsych evaluation prior to testing?* |
| ·       *comparing patients to pre diagnostic testing for diagnosis is not helpful as these numbers generally dont exist, more comprehansive neuropsychology testing allows for determination of likely premorbid function and in these settings such diagnostic algorithms can be helpful in the setting of significant cognitive impairment results often need to be discussed with the primary carer for patient safety* |
| ·       *Psychiatric evaluation, whilst possibly useful, is often difficult to obtain in both metropolitan and regional/ rural areas and when available there is a long wait which often makes it not practical.* |
| ·       *It is essential that all the treating team and carers are aware of significant decline in cognitive functioning to ensure appropriate and person-centred care is delivered* |
| ·       *In Sections 3.4 and 3.5, while the use of PET may be beneficial, making a diagnosis contingent on ruling out PET-related abnormalities, particularly those that could suggest AD (e.g. PIB scan) is problematic from a feasibility point of view. This is particularly true where individuals may live in areas where there is not ready access to a PET scanner or there are financial or other issues that may impact access to resources. This could have the unintentional result of unnecessarily delaying diagnosis and treatment trials.* |
| ·       *In regards to the radiological examinations mentioned in items 3.4 and 3.5, people with PD who live regionally, rurally and remotely are unlikely to be able to access the examinations without travel. The burden of travel for the examinations must be considered against their utility.* |
| ·       *Amyloid PET are not readiy available, and would not necessarily help with excluding a diagnosis of dementia in PD as studies have identified that there is often Alzheimer pathology in dementia with PD.* |
| ·       *3:12- the wording is unclear. If a client/patient consents and undergoes testing then yes they should receive feedback unless they specifically request that do not want to know the outcome 3:13- this is patient dependent but yes providing information on ; Above, at, or below expectations or level of impairment for tasks/domains should be discussed* |
| ·       *3.12 and 3.13 I suppose you can say it's consent or I would consider it an invitation for patient to learn about the results and possible strategies. Some people aren't interested in knowing so I don't think we should impose on them. But if there are sig deficits or impairment I would try to convince them to come back for results and let me share with sig others who are worried about them. I think the format and language used in feedback should be tailored to the individual levels as well.* |
| ·       *I disagree with recommendation 3.13, as cognitive change can be a significant source of worry for those with PD, even when no subjective cognitive complaints are currently present. Bench marking their performance against normative data may lead to increased anxiety.* |
| ·       *feedback should focus on outcomes - what the best course of action is for monitoring and managing the symptoms with an emphasis on quality of life and activities of daily living.* |
| ·       *I think the framing of the information needs to be done very carefully. Rather than talk about averages I would focus on the functional aspects of what the results are telling us.* |
| ·       *I think transparency is important, and many people with PD feel more empowered with information about their condition. This is also the case for family, who are often overlooked in the process. They need information on what they may expect.* |
|  |
| **Neuropsychological evaluation** |
| ·       *Please note that I currently only administer neuropsychological testing or other forms of cognitive assessment in a research capacity, and not for diagnostic/prognostic purposes. For cases where subjective cognitive decline is perceived by the individual, informant or clinician, a brief cognitive screen should be performed prior to referral for comprehensive cognitive assessment. If behavioural or psychological symptoms are present, I think that referral to a psychologist and ruling out of other potential diagnoses should occur prior to referral for comprehensive cognitive assessment. Performing a brief cognitive screen in these individuals (as per above), however, would be appropriate. In cases of diagnostic uncertainty, referral for comprehensive cognitive assessment on its own is not likely to be informative in resolving the query, and if it occurs, should be paired with additional testing (e.g. blood tests and neuroimaging). In cases where the individual with PD requests a comprehensive cognitive assessment, I don't this referral should be provided without some evidence of current cognitive change, either self-reported or as indicated by a screening. This may otherwise be a significant source of anxiety for the individual and result in multiple unnecessary referrals. That being said, a baseline cognitive evaluation at time of diagnosis would be very beneficial for tracking future change.* |
| ·       *4.3 Neuropsychology only, OT are not trained to deliver comprehensive neuropsychological evaluations.* |
| ·       *The decision as to whether to refer a patient for a comprehensive neuropsychological assessment will be influenced by whether or not the patient and their family would like to be referred for an assessment as well as the i) types and ii) degree of cognitive impairments and whether (i) and (ii) are affecting basic and instrumental ADLs.* |
| ·       *4.3 80% of Pd decline, we can't test every person just to detect that decline. the clinician can do short screens to detect CI. comprehensive evaluation required when there is questions regarding domains of cognition effected, Dx is unclear or where surgical interventions are being considered. etc* |
| ·       *note some geriatricians and neurologists perform these evaluations as part of their usual clinical practice, additional referrals not always necessary. in absence of these evaluations being available from usual treating physicians, allied health input for comprehensive assessment is highly valuable.* |
| ·       *Comprehensive cognitive evaluation is useful and ideal, however may not be required for all individuals with PD. It would be most beneficial if there is uncertainty regarding diagnosis and/or whether there is a specific question to be asked, and if it will guide further treatment/ therapy.* |
| ·       *4.2 -for behavioural and psychological symptoms, referral to psychiatrists and psychologists should be considered first before neuropsychology - these symptoms can exacerbate cognitive symptoms so ideally should be optimised first before we test them. Capacity assessment is not about cognition alone and should be evaluated by doctors in conjunction with allied health looking at functional abilities and specific decisions - neuropsychological assessment is not necessary or adequate on its own. Neuropsychological assessment and driving has modest correlation so should not be referred for the sole reason of driving. 4.3 - once again, people with PD should be referred for cog screen at the start and perhaps on a regular interval but only when there are more concerns re diagnostic clarification, or they are very high functioning and the screen is not capturing the extent of the cog deficits should they be referred to NP* |
| ·       ***Reason for referral****: When other potential risk factors for cognitive decline are also present (e.g. positive for ApoE4 allele; history of TBI or significant pesticide exposure)* |
| ·       *4 - For a clinical assessment, ADL and IADL can be elicited during the interview and a questionnaire is not necessary in my opinion.* |
| ·       *For IADL a good clinical history and collateral history may be sufficient* |
| ·       *the challenge with many of these scales in PD is often they are abnormal with early cognitive changes/ MCI and may not have the capacity to measure worsening over time there is always the influence of dopaminergic state and fatigue on performance to consider in interpreting results* |
| ·       *There are also a range of other tests that are highly effective and sensitive to cognitive decline (Stockings of Cambridge, similarities, Rivermead paragraph). The global assessment is often used inappropriately - many people with MCI can score very well on MOCA and MMSE.* |
| ·       *Addenbrookes is an excellent test for consideration.* |
| ·       *4.13- Rey Figure would be challenging for a portion of patients - it can be frustrating for patients and there are just too many details. Also, Rey fig has too much executive function component so it's not really a pure visual memory measure anyway, hence I would not use it. BVMT is a possible nice alternative of visual memory but my experience of using BVMT is that people may do worse on BVMT than on WMS Logical Memory, becasue they get overwhelmed and struggle to look at 6 designs at the same time whereas LM is only 1 stimulus at a time. Also the scoring of BVMT is more stringent than LM so this is another important consideration.* |
| ·       *While I interpret the results of neuropsychological tests and other cognitive tests, it is for research purposes only and not in a research capacity. Regarding recommendation 4.9, I believe that testing should be conducted in both the ON and OFF state, to fully assess cognitive change, as some individuals do show at least a modest improvement in cognitive function in response to medication. If it is not possible to do both due to time or resource constraints, then I agree that testing should be performed in the ON state, unless specifically indicated to do otherwise.* |
| ·       *People with PD should be instructed to take their dopaminergic medication 30 minutes to an hour prior to neuropsychological assessments to induce the ON- state - ideally the appointment should be scheduled around medication routine, and not the other way around* |
| ·       *4.14.10 - Every patient is different in terms of how the medications affect them. Ax should be done when they are "ON" but sometimes that means sig dyskinesia for our patients. Thus, I don't believe in asking patient to change the timing of taking medications for the sake of the assessment. A lot of patients would take at least 1-2 doses of medications during the assessment and when they stiffen up or tremors or dyskinesia get worse, we can adapt the assessment to suit them - eg, we might choose to take a break, or do verbal tasks that don't require motor function, and change the order of the testing battery flexibly* |
| ·       *Q4.10- often not onpractical due to patients medication schedule and length of comprehensive neuropsych assessment (2.5-3 hours). The impact of on-off on cognition has also been demonstrated to vary by motor symptom and disease duration. Care should be taken to mention any potential influence and state whether in or off- particularly when re-assessment is required.* |
| ·       *People with PD are at a distinct disadvantage when it comes to best-practice PD management. Accessibility issues must be considered.* |
| ·       *Should be face to face assessments because of difficulty in doing cognitive assessments via telehealth* |
| ·       *Face to face assessments strongly recommended for validity and comprehensive assessment* |
| ·       *My key concern would be availability and access to comprehensive cognitive evaluation. Even with telehealth assessments being made available, are there any neuropsychologists available to deliver this level of care.* |
| ·       *it is important for all patients no matter where they live to have access to neuropsychological assessment* |
| ·       *4.4 - definite challenges with remote assessment methods - don't feel this is able to capture the extent of deficits accurately. Would always have preference for face to face.* |
| ·       *4.4-telehealth NP Ax is challenging for people with dysphonia, dysarthris, and tremors - I would only conduct Ax in person to account for impact and make appropriate adjustment to how the tests should be administered, what alternative tests should be used, as well as interpretation* |
|  |
| **Post-diagnostic care** |
| •       *Guanfacine for treatment of dementia and MCI* |
| •       *management of cognitive symptoms often needs to be balanced against the need for medications to treat motor symptoms and individual patient preferences/ carer availability etc needs to be part of the decision making process* |
| •       *Antipsychotics should be considered in individuals with cognitive impairment with problematic symptoms, such as hallucinations, but are not required for all individuals with cognitive impairment. Device assisted therapy should be considered if indicated based on motor symptoms and fluctuations, not based on cognition.* |
| •       *Invasive therapies are hard for people with dementia* |
| •       *This needs to be decided case by case* |
| ·       *Non-pharmacological treatments: Cognitive Behaviour Therapy, Mindfulness, Gentle exercise, Fitness increasing aerobic exercise, boxing; progressive resistance training* |
| ·       *Lifestyle modifications: Healthy diet and risk reduction (e.g. lower BMI, lower cholesterol, improved heart health, etc.), Other changes to lifestyle, psych review for mood, exercise physiologist, Not required for all but if have dysphasia refer to ST etc., the advice should match the need.* |
| ·       *The section on the previous page for access to professionals - this would depend on the type of cognitive disorder diagnosed. For 5.12 - This might depend on the specific cognitive deficits identified and subjective reports of driving ability. For some of these questions, I would imagine these post-diagnostic support options/considerations could not be completed in a single session.* |
| ·       *The provision of post diagnostic care plans and referral to services is beyond the resources of average outpatient appointment. The provision of a care navigator post diagnosis would have a major impact on the person with PD's wellbeing, and reduce caregiver burden.* |
| ·       *note i believe driving, advanced care and life planning should be discussed with ALL patients with parkinsons disease preferably prior to development of cognitive concerns, then rediscussed regularly particularly at the time of any significant clinical change such as development of further cogntive decline, physical decline, aging or dementia* |
| ·       *The last section - items that I neither agree or disagree with are too directed - ech person may differ and may not need this specific recommendation. Recommendations whould be personalised for each individual based on their cognitive profile.* |
| ·       *Recommendations and further assessment need to be tailored to the individual client* |
| ·       *Failure to ensure an advanced care directive is in place early on in the person's trajectory results in multiple difficulties as their condition deteriorates and places unnecessary stress on family members and may often result in inappropriate or futile treatments.* |
| ·       *Cognitive coping strategies are very helpful for some - but not all. Some people find they increase stress esp if they have new diagnosis. Respond to the needs of the patients as they arise* |
| ·       *5.12, 5.13, 5.14 and 5.16 - I think for MCI patients, it is a delicate process to discuss driving, ACP and support/adjustment, so it should be determined on a case by case basis and taking into consideration of individual psychosocial situations and their psychological wellbeing. You don't want to make some of these patients more anxious. These issues should be raised at some point but the timing is the key and should not be a blanket approach.* |
| ·       *management should be tailored to the needs of the PLWP and the issues that related most to the activities of daily living and QoL.* |
|  |
| **Additional Comments** |
| •       *Suggest cognitive behaviour therapy for anxiety and depression.* |
| •       *You've done a great job capturing the current state of the field. The field, however, very much needs greater investment into the development of pharmacological and non-pharmacological strategies tailored specifically to address cognitive impairment in PD.* |
| •       *People with PD need to have appropriate motor and non-motor symptom assessment by their treating team. This is likely best done via the specialist, general practitioner and/ or PD nurse.* |
| •       *Some responses would be different if a diagnosis of Lewy Body dementia rater than PD associated dementia were being entertained. In these patients I would suggest a cholinesterase inhibitor. Not sure if the wording of this issue would help in the form design* |
| •       *Assessing cognitive decline in patients with PD can be challenging. There is a need for further national guidelines around cognitive assesmsents of patients with PD and related disorders e.g., LBD, PSP and CBD* |
| •       *Keep guidelines as "working with" the person with PD and their carer/family. Not "doing to" without consent/agreement.* |
| •       *I think that cognitive assessment should occur at regular intervals, regardless of whether a person expresses a subjective reduction in cognition. It should be a regular occurrence so that any decline can be detected early and supports put in place.* |
| •       *Addressing/considering inequity of services for people with PD and their caregivers in regional, rural and remote locations.* |
| •       *Great idea to formalise this!* |
| •       *Stronger emphasis on planning ahead. It is not uncommon for a GP to tell a patient who requests referrals to appropriate services to be told, oh things aren't that bad yet, e.g. referral to speech pathologist and exercise physiologists.* |
| •       *Having a carer/ support person present to promote understanding* |
| •       *Provide increased opportunities for social interaction.* |
| •       *There's a lot of qualitative and observational data involved with PD patients so putting outside the motor and speech challenges, i think telehealth is going to be challenging. I think efforts should be made to increase access to trained clinicians for face-to-face assessment. First step is of course to do more regular cog screening and only refer to neuropsychologist when needed - that's probably the main thing to include in the guideline.* |

| *eTable 3*. Written feedback from Round 2 |
| --- |
| **Who should receive a neuropsychological evaluation?** |
| *·      I disagree that individuals who show impairment in global cognitive function on a brief screen are less likely to tolerate or benefit from comprehensive neuropsychological assessment. I think that such assessment is critical to understand the specific domains of cognitive function impacted (i.e. cognitive subtype), which is directly relevant to diagnostic criteria for cognitive impairment in PD and can inform tailored treatment plans. It is also critical for tracking progression of cognitive impairment over time.* |
| *·      For question 2.8 - I think this would depend on the purpose of assessment, and I don't know if their MOCA score alone is sufficient to answer whether a patient would benefit from/tolerate more comprehensive cognitive assessment. Similarly, if the question is around differential diagnosis and their MOCA is say 19/30 for example, a more comprehensive evaluation might still be useful.* |
| *·      One needs to take into account why one is undertaking neuropsychological assessments e.g., help assess capacity.* |
| *·      I disagree with 2.8 as I feel that understanding more about the cognitive deficits through neuropsychological exam may help to properly stage and guide treatment for someone, which the screening measure outcome can't really give.* |
| *·      Comprehensive neuropsychological assessment may assist in confirming the diagnosis in the early stages allowing for targeted interventions to be commenced. Neuopsych assessment is not required for significant impairment as the memory score is self-explanatory. Repeating the assessment is over servicing and there is nothing to be gained, the patient is unlikely to tolerate the assessment.* |
| *·      Neuropsychological testing should be reserved for patients with early or mild cognitive impairment* |
| *·      2.12- Agree but sometimes a different form of neuropsych assessment may be needed e.g. capacity, targeted etc.* |
| *·      Question 1 - I think if there are subjective cognitive decline supported by collateral reports of functional decline, and if they score higher than 20 on the MoCA they should be referred for comprehensive neuropsychological assessment. Not the other way round.* |
| *·      Assessments need to match the available symptom-based interventions to provide the best QoL outcomes for the patient and their care team.* |
|  |
| **Diagnosis** |
| *·      Regarding recommendation 3.8, I think that it's important that the person with PD and their designated care partner should both receive verbal and written information regarding the appointment, to help ensure understanding of the process. Regarding recommendation 3.10, I think that this needs to be more specific, in order to provide more tailored guidance.* |
| *·      3.8 - not sure what you mean by "prepare" for assessment 3.9 - is there are reason you have selected counsellor over psychologist. Whether or not this is appropriate would also appear to depend on type of "distress" - are you referring to emotional distress (in which case referral to a psychologist may be appropriate regardless of wait-times.* |
| *·      The context of feedback and data/ information sharing can include patient / caregiver consent however there may be situations whereby medical law compels further liaison with health professionals in the best interest of the patient +/- caregiver. I.e. Duty of Care* |
| *·      For 3.7. This should be completed with consideration of motor impairment as well. It should not be an either / or assessment for cognitive / motor.* |
| *·      3.7. Consider self-report tools, informant interview, observational evaluation and formal testing for assessment of Activities of Daily Living (ADL) to determine functional impairment due to cognitive impairment IN ADDITION To motor impairment.* |
|  |
| **Neuropsychological evaluation** |
| ·      Yes, this might help to assess visuospatial/visuo-motor based functions for example |
| ·      it may depend also on disease state and stage as well as the presence of symptom fluctuations |
| ·      the difficulty is that optimal function in relation to levodopa medication is u-shaped: too little or too much result in poor cognitive scoring. Cools R, D'Esposito M. Inverted-U-shaped dopamine actions on human working memory and cognitive control. Biol Psychiatry. 2011 Jun 15;69(12):e113-25. doi: 10.1016/j.biopsych.2011.03.028. Epub 2011 May 4. PMID: 21531388; PMCID: PMC3111448. |
| ·      This is challenging due to issues of symptom fluctuations. I strongly agree with statement 4.10 provided that the symptoms are being optimally treated - it may be difficult to evaluate whether this is the case. If there are significant periods of the day where symptoms are not being adequately managed, this will also be reflected in their altered cognitive functionality and impacts on ADLs. |
|  |
| **Post-diagnostic Care** |
| ·      Regarding 5.11 and 5.15, I disagree that time of diagnosis is the most appropriate time to have these discussions with people with PD and their caregivers. I think that adequate time should be allowed to process the diagnosis, and a follow-up appointment should be scheduled in order to discuss driving fitness/capacity and advance care planning/directives and estate planning. |
| ·      whilst advanced care and estate planning is important, I disagree that it needs to be done AT the time of diagnosis of any cognitive disorder. The term cognitive disorder does not reflect the diagnostic etiology (e.g. delirium vs dementia, etc) , severity nor sensitivity when it comes to sensitive discussions on this topic. My personal view is the timing of this discussion will be situational. |
| ·      discussions about future planning are best undertaken prior to the diagnosis of cognitive impairment but should be commenced if cognitive impairment is suspected. |
| ·      These are important discussions but there's also a lot of information for patients and families to process at the time of diagnosis. These areas should be prioritised and discussed but may require a few appointments. |
| ·      Advance care planning / directives and estate planning should be discussed with people with PD and their care partners - however not necessary at the same time as the diagnosis of a cognitive disorder. These issues should be discussed if not at the time of diagnosis, then shortly thereafter. |
| ·      5.4 should also include exercise physiologists as an accredited health service provide. |
|  |
| **Additional comments** |
| ·      The topic on deprescribing is important but must be handled with care. It should ideally be part of joint care involving a specialist or physician with sufficient experience in the management of Parkinson's and other potential comorbidities. |
| ·      The differentiation of dementia with Lewy Bodies and Parkinsons related dementia is difficult but important in terms of therapeutic options. The answers to some of these questions would differ slightly depending on the dementia subtype diagnosis |
| ·      Post-diagnostic care should involve GP, specialist, neuropsychologists, carers and other family members. We need to think about "individualised" based approaches for the diagnosis and evaluation of patients. |
| ·      Important questions - driving and ACP are critical and not done enough by clinicians. |
| ·      Cognitive disorders should also be considered in conjunction with motor disorders as the two are interlinked. |
